# Supplementary material for: Comparison of the Anti-Tumour Activity of the Somatostatin Receptor (SST) Antagonist [177Lu]Lu-Satoreotide Tetraxetan and the Agonist [177Lu]Lu-DOTA-TATE in Mice Bearing AR42J SST2-Positive Tumours
Source: Pharmaceuticals (Basel). 2022 Aug 30;15(9):1085. doi: 10.3390/ph15091085 (PMC9506113; doi:10.3390/ph15091085)
Supplement: Supplementary file 1 [file pharmaceuticals-15-01085-s001.zip › pharmaceuticals-1812909-supplementary.pdf]

Table S1 Proportion of xenografted AR42J tumours showing relapse or senescence at end of study

**Treatment outcomes**	**Vehicle (n=13)**	**[^{177}Lu]Lu -DOTATATE 15 MBq (n=18)**	**[^{177}Lu]Lu -DOTATATE 30 MBq (N=18)**	**[^{177}Lu]Lu -satoreotide tetraxetan 15 MBq (N=13)**
Mice with complete tumour relapse	13/13 (100)	11/15 (73)	7/15 (47)	1/10 (10)
Mice with tumour relapse and senescent tumour lobules	0/13 (0)	4/15 (27)	6/15 (40)	7/10 (70)
Mice with complete tumour senescence	0/13 (0)	0/15 (0)	2/15 (13)	2/10 (20)

Data are expressed as n/N (%).

Table S2. Radioactivity uptake of ^{177}Lu-DOTATATE and ^{177}Lu-satoreotide tetraxetan in different organs at 96 hours post-administration of last treatment, and its relationship with SST2 expression level

Treatments	Tumour	Kidneys	Adrenals	Femur	Spleen	Tail
SST2 expression level determined by IHC	High	None	Moderate	Minimal	Minimal	None
**[^{177}Lu]Lu-DOTATATE 15 MBq**	1.0 (0.30)	0.066 (0.012)	0.11 (0.015)	0.006 (0.00051)	0.0032 (0.00026)	0.01 (0.0022)
**[^{177}Lu]Lu-DOTATATE 30 MBq**	2.2 (0.22)	0.20 (0.043)	0.23 (0.018)	0.008 (0.00043)	0.006 (0.00057)	0.016 (0.0037)
**[^{177}Lu]Lu-satoreotide tetraxetan 15 MBq**	3.5 (0.14)	0.51 (0.077)	0.12 (0.061)	0.014 (0.0014)	0.013 (0.0013)	0.015 (0.0014)

Data are expressed as mean (standard error of the mean) in MBq/g of tissue. IHC, immunohistochemistry; SST2, somatostatin receptor subtype 2.

Table S3. Mean haematological parameters at 96 hours post-administration and at the end of study of ^{177}Lu-DOTATATE and ^{177}Lu-satoreotide tetraxetan, in comparison to the vehicle control group

	**RBC (10^3/mm^3)**	**Ht (%)**	**Hb (g/dL)**	**Platelets (10^3/mm^3)**	**WBC (10^3/mm^3)**	**Lymphocytes (10^3/mm^3)**	**Neutrophils (10^3/mm^3)**
At 96 hours post-administration of last treatment							
**Vehicle**							
Mean	7.7	40	12	288	3.1	1.9	1.0
**[^{177}Lu]Lu-DOTATATE 15 MBq**							
Mean	8.0	43	12	283	2.1	1.2	0.77
Percentage of variation	3.2	6.4	-1.4	-2.0	-32	-37	-26
**[^{177}Lu]Lu-DOTATATE 30 MBq**							
Mean	8.4	46	13	292	2.6	1.3	1.1
Percentage of variation	9.0	14	5.0	1.3	-16	-29	2.6
**[^{177}Lu]Lu-satoreotide tetraxetan 15 MBq**							
Mean	8.5	51	13	258	2.2	1.0	1.0
Percentage of variation	11	25	12	-11	-29	-44	-8.7
End of study							
**Vehicle**							
Mean	7.3	39	11	282	2.5	1.2	1.1
**[^{177}Lu]Lu-DOTATATE 15 MBq**							
Mean	7.3	40	11	352	4.1	2.8	1.1
Percentage of variation	-0.6	5.2	-1.9	25	64	129	3.3
**[^{177}Lu]Lu-DOTATATE 30 MBq**							
Mean	7.5	38	11	400	3.9	2.3	1.3
Percentage of variation	1.8	-0.7	-2.4	42	56	95	20
**[^{177}Lu]Lu-satoreotide tetraxetan 15 MBq**							
Mean	8.4	43	13	347	5.3	3.9	1.2
Percentage of variation	14	12	14	23	113	229	4.9

Percentage of variation compared to the vehicle control group. Hb, haemoglobin; Ht, haematocrit; RBC, red blood cell count; WBC, white blood cell count.

Table S4. Treatment-related microscopic observations at 96 hours post-administration of last treatment and at the end of study

Treatments	Vehicle	[^{177}Lu]Lu - DOTATATE 15 MBq	[^{177}Lu]Lu - DOTATATE 30 MBq	[^{177}Lu]Lu - satoreotide tetraxetan 15 MBq
At 96 hours post-administration of last treatment				
Number of examined mice	3	3	3	3
**Bone marrow**				
**Myeloid/erythroid ratio**	**3**	**3**	**3**	**3**
Moderate increase	1	2	3	3
Mild increase	2	1	-	-
Mean score*	***2.3***	***2.7***	***3.0***	***3.0***
**Spleen**				
**Extramedullary haematopoiesis**	**3**	**3**	**3**	**3**
Marked decrease	2	-	-	-
Moderate decrease	1	2	1	1
Mild decrease	-	1	2	2
Mean score*	***3.7***	***2.7***	***2.3***	***2.3***
**Kidneys**				
**Basophilic tubules**	**0**	**2**	**1**	**1**
Minimal	-	2	1	1
End of study				
Number of examined mice	10	11	11	7
**Bone marrow**				
**Myeloid/erythroid ratio**	**9**	**11**	**11**	**7**
Moderate increase	-	1	3	6
Mild increase	3	6	6	-
Minimal increase	6	4	2	1
Mean score*	***1.2***	***1.7***	***2.1***	***2.7***
**Eosinophilic vacuolated cells**	**0**	**0**	**1**	**1**
Mild	-	-	1	-
Minimal	-	-	-	1

*Mean score is Σ number of animals x severity score / number of examined animals in the group.

Figure S1

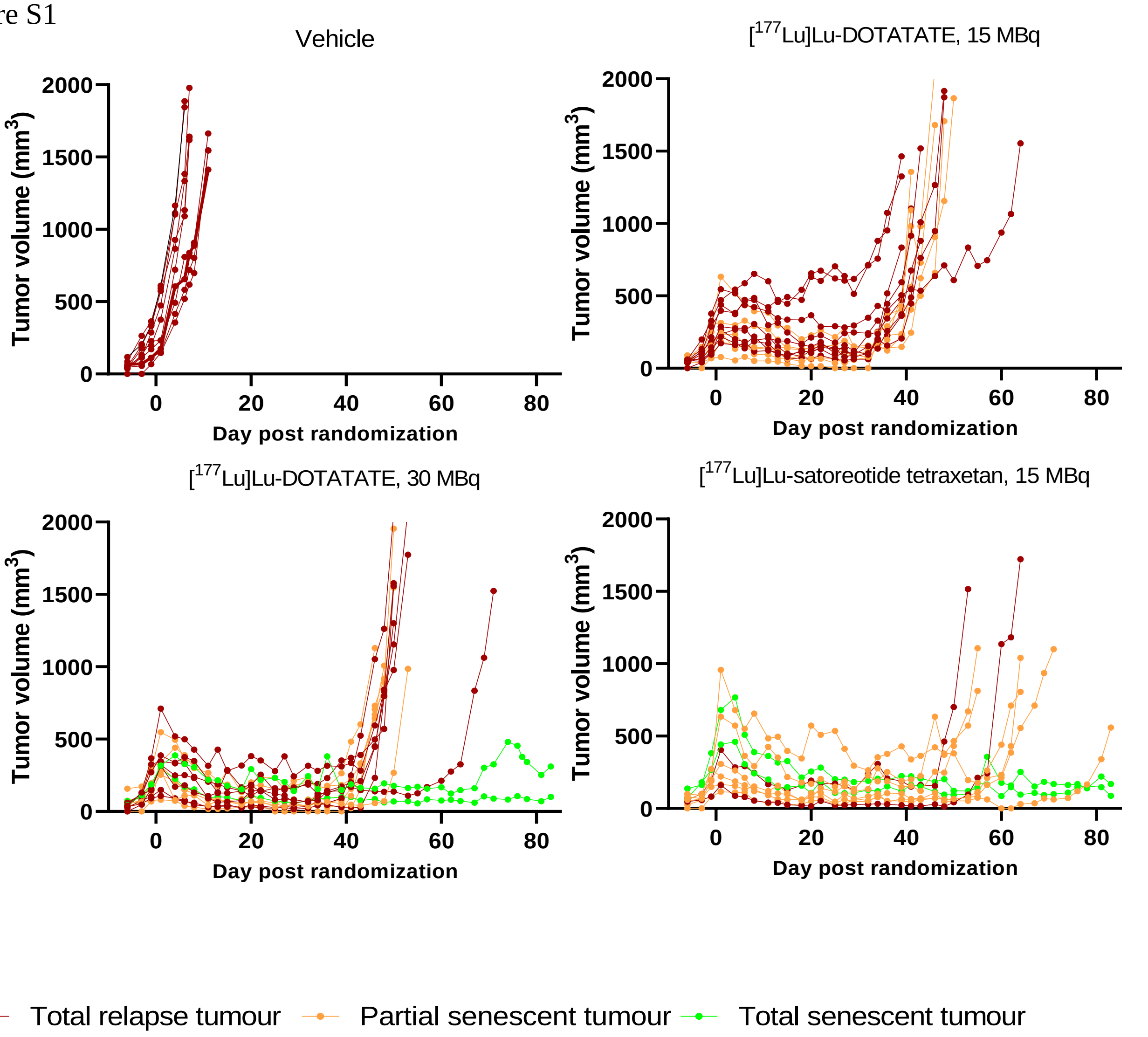

Figure S1. Individual Tumour growth over time of Vehicle, [^{177}Lu]Lu -DOTATATE at 15 and 30 MBq and [^{177}Lu]Lu -satoreotide tetraxetan at 15 MBq
